# Supplementary figures and images for: A multivariate, quantitative assay that disentangles key kinetic parameters of primary human T cell function in vitro
Source: PLoS One. 2020 Nov 9;15(11):e0241421. doi: 10.1371/journal.pone.0241421 (PMC7652339; doi:10.1371/journal.pone.0241421)

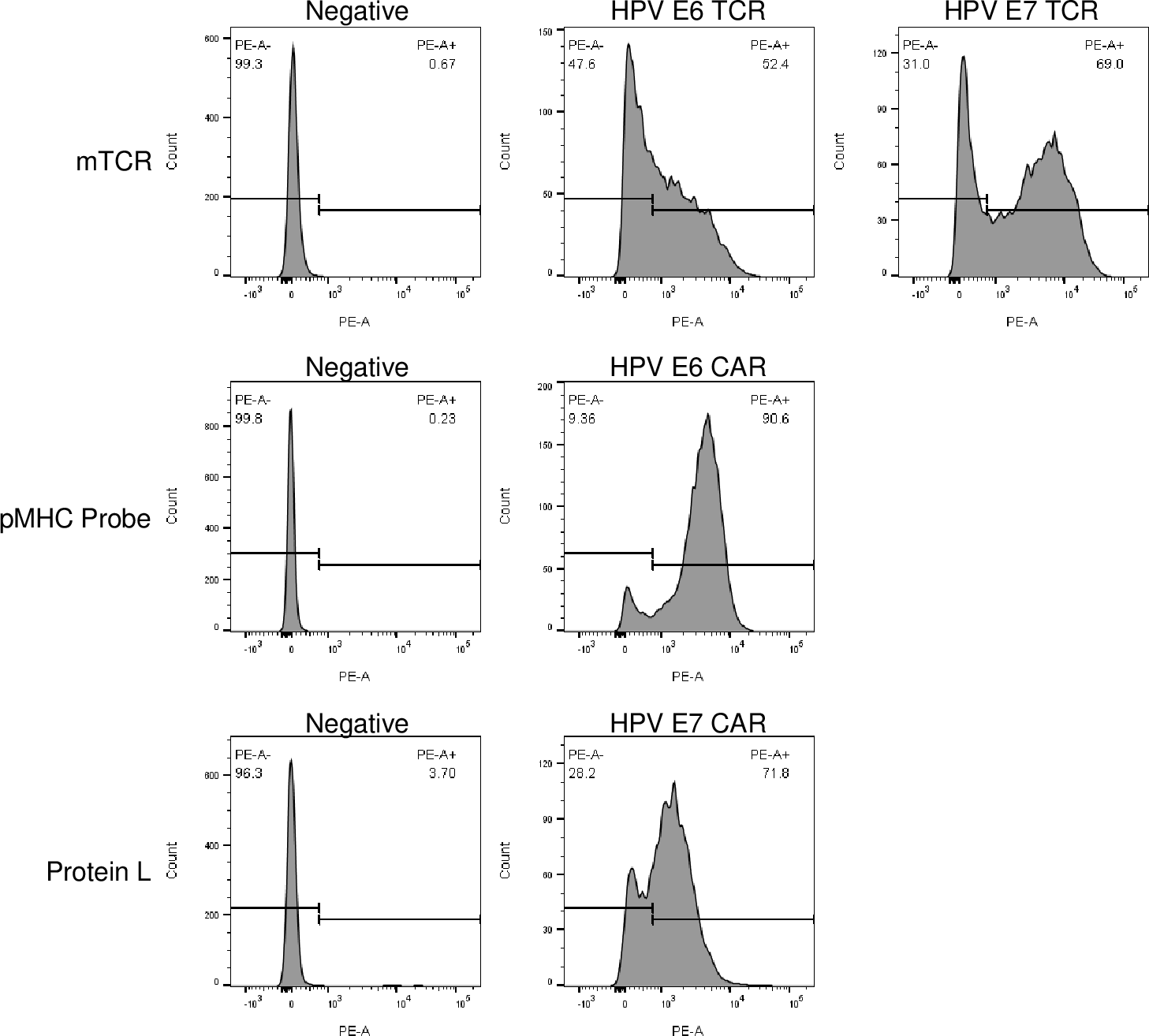

Supplement: S1 Fig — (TIF) [file pone.0241421.s001.tif]

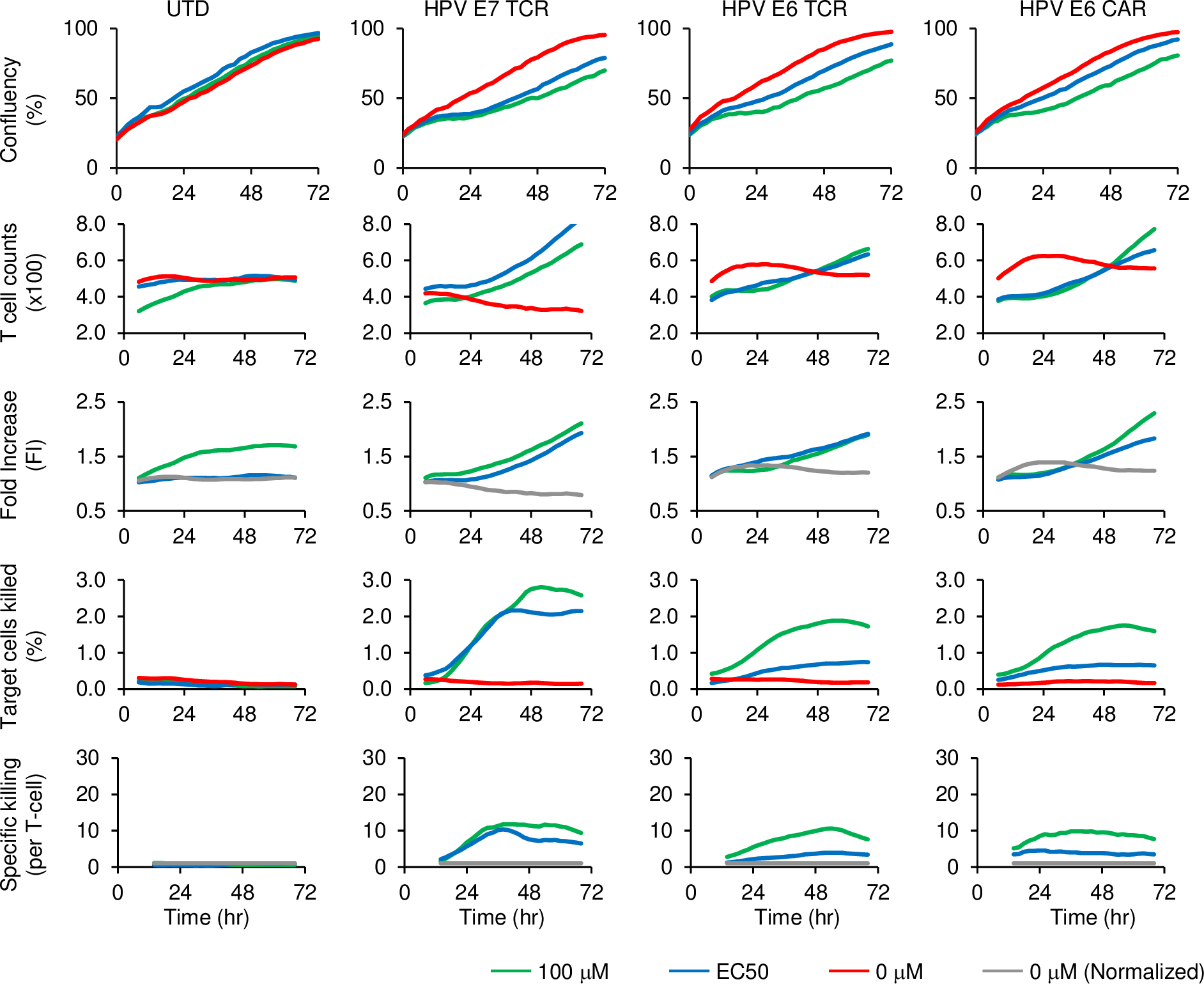

Supplement: S2 Fig — Graphs of target cell confluency, T cell counts, relative T cell proliferation, percentage of target cells killed, and specific target cell killing per T cell are summarized here. Target cells are loaded with peptide at 100 uM (green), 0 uM (red), or a concentration similar to their EC50 (blue). (TIF) [file pone.0241421.s002.tif]

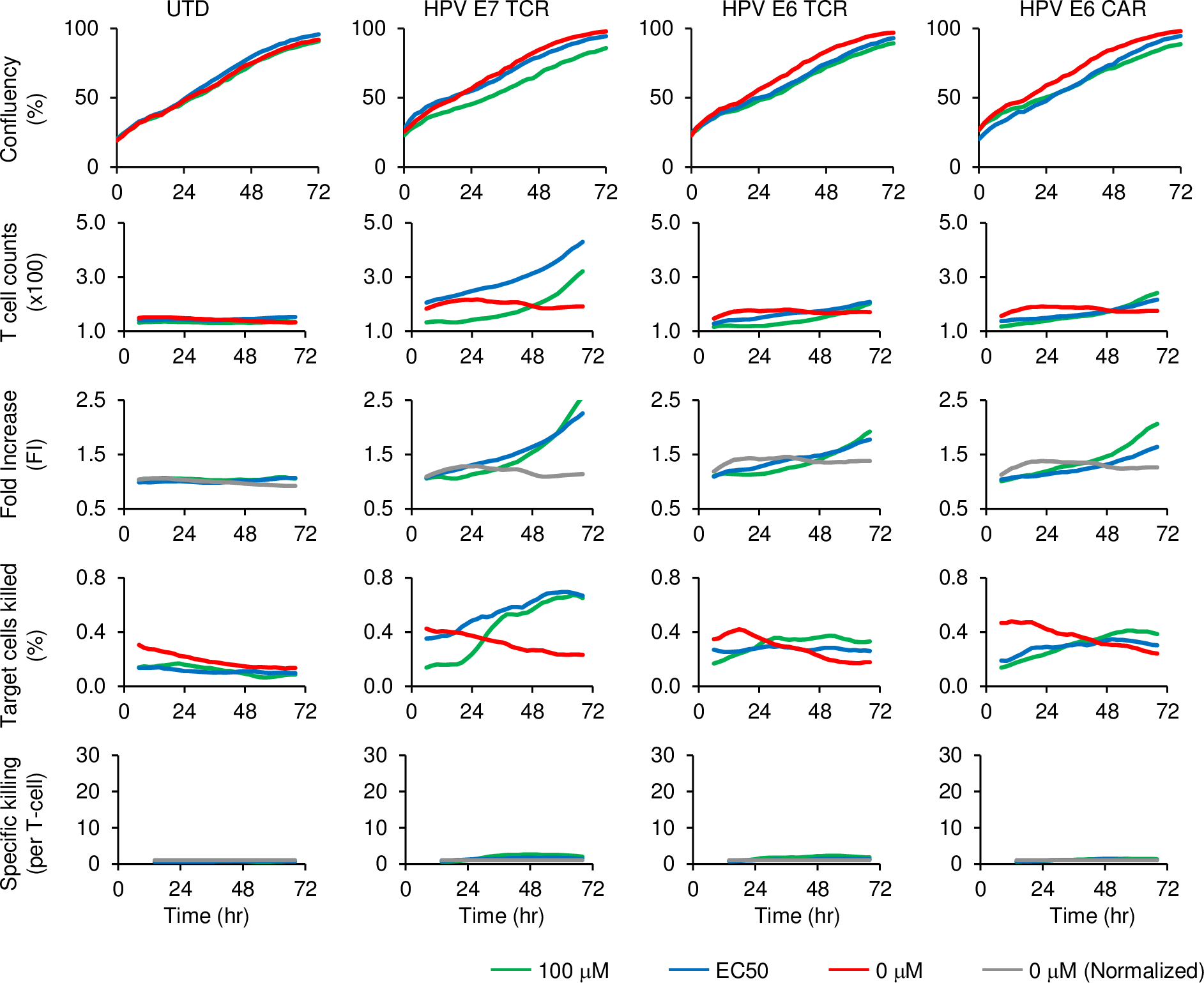

Supplement: S3 Fig — Graphs of target cell confluency, T cell counts, relative T cell proliferation, percentage of target cells killed, and specific target cell killing per T cell are summarized here. Target cells are loaded with peptide at 100 uM (green), 0 uM (red), or a concentration similar to their EC50 (blue). (TIF) [file pone.0241421.s003.tif]

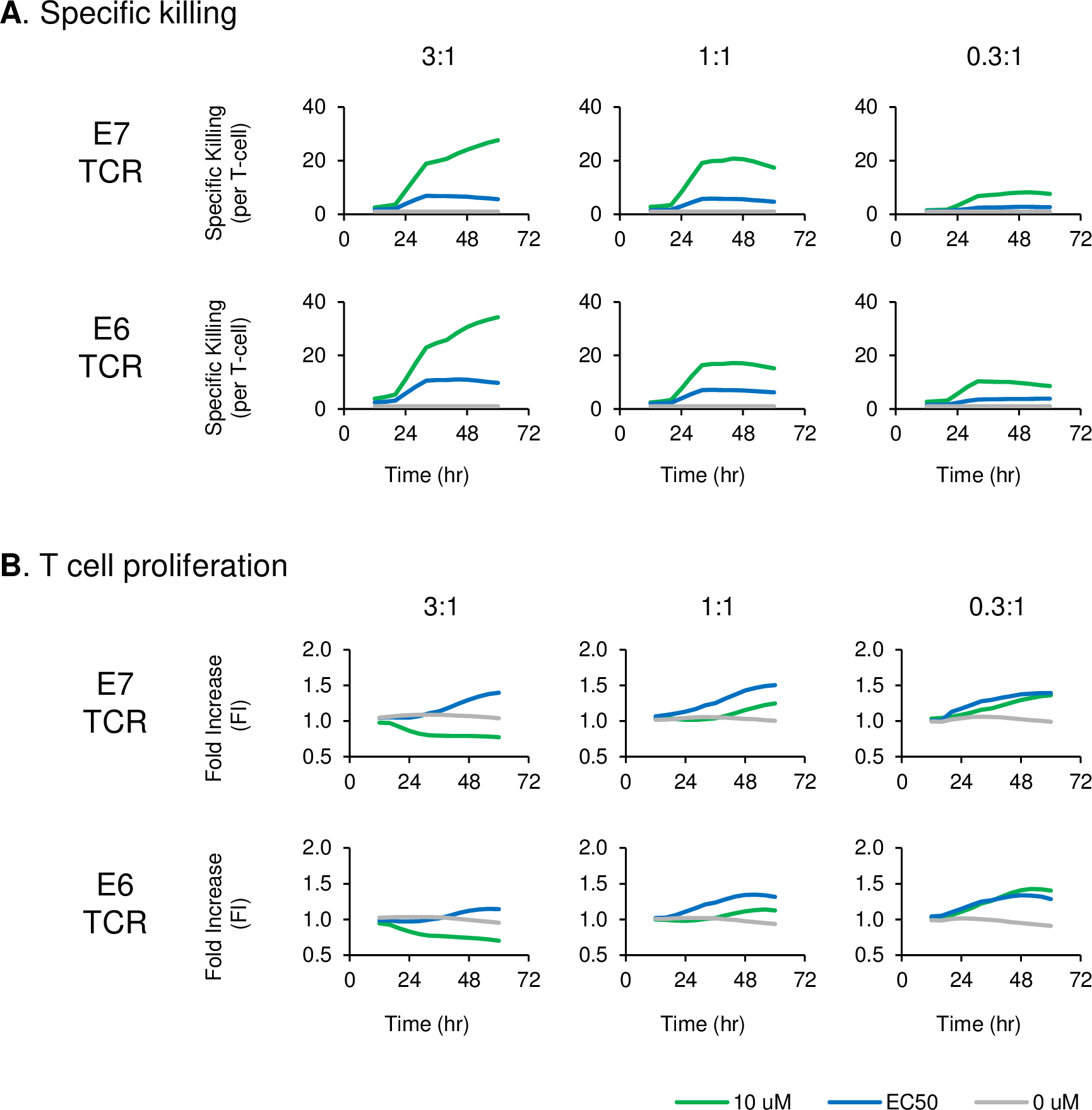

Supplement: S4 Fig — Graphs of relative T cell proliferation and specific target cell killing per T cell from three different E:T ratios are summarized. Target cells were loaded with peptide at 10 uM (green), 0 uM (gray), or a concentration similar to their EC50 (blue). (TIF) [file pone.0241421.s004.tif]

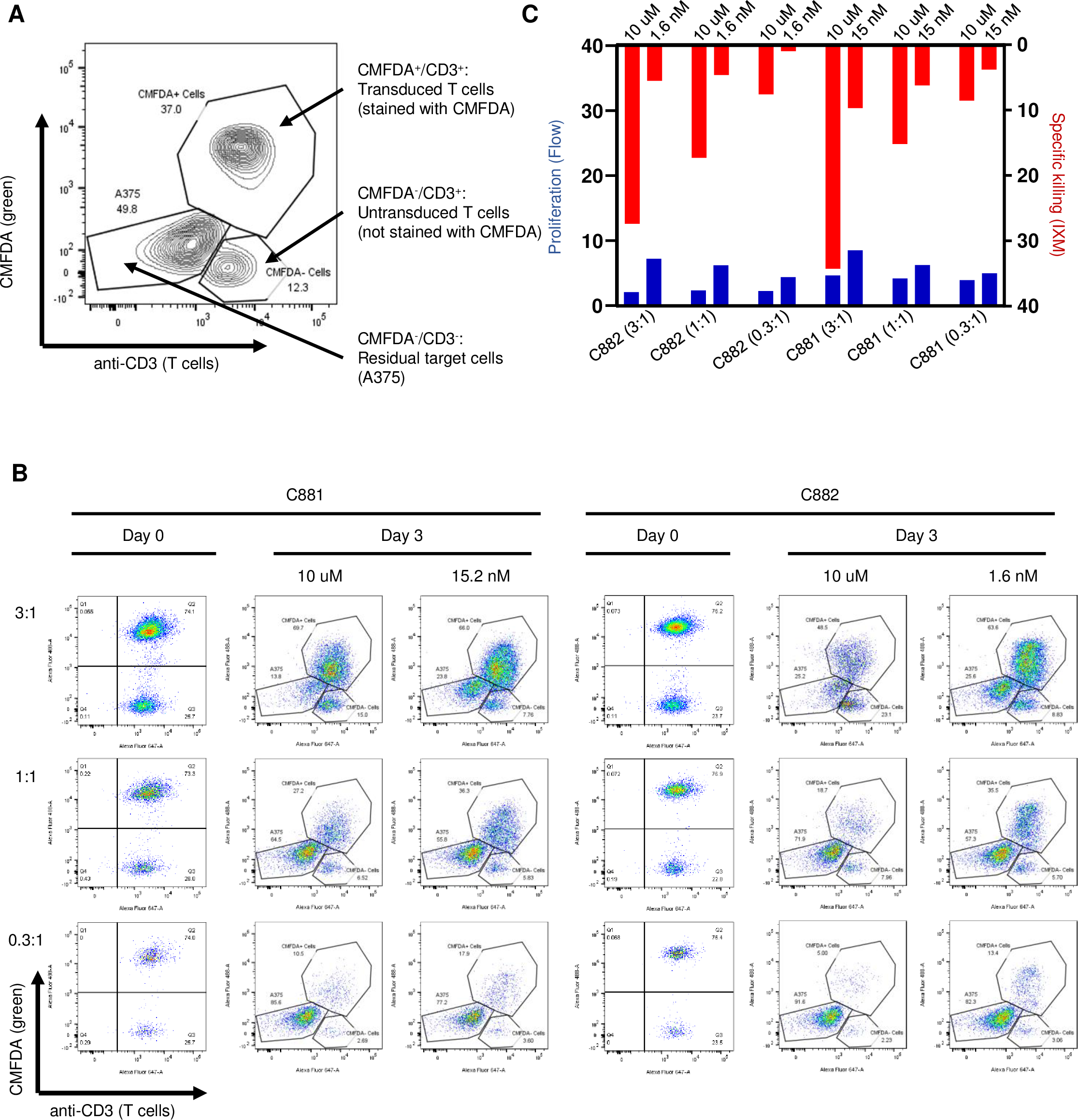

Supplement: S5 Fig — Method and results of determining T cell proliferation are summarized (see Materials and methods for further details). A. An example contour plot to illustrate how TCR or CAR-transduced T cells were separated from the untransduced T cells and target cells. B. Scatter plots of T cell flow cytometry at day 0 and day 3. C. Comparison of specific killing from IXM (red) and T cell proliferation (blue) determined by flow cytometry revealed an inverse relationship between cytotoxicity and T cell proliferation. (TIF) [file pone.0241421.s005.tif]
