# Supplementary material for: A multivariate, quantitative assay that disentangles key kinetic parameters of primary human T cell function in vitro
Source: PLoS One. 2020 Nov 9;15(11):e0241421. doi: 10.1371/journal.pone.0241421 (PMC7652339; doi:10.1371/journal.pone.0241421)
Supplement: Attachment — Submitted filename: Response to reviewers.docx [file pone.0241421.s006.docx]

Dear Dr. Gangopadhyay and/or other editor(s):

Please see the following for details of our reponses to editor and reviewers’ comments. In particular, we listed our funding and competing interests statement statement as requested.

Funding statement:

The funder provided support in the form of salaries for authors GLH, DPN, GBG, KRN, AK, and HX, but did not have any additional role in the study design, data collection and analysis, decision to publish, or preparation of the manuscript. The specific roles of these authors are articulated in the ‘author contributions’ section.

Competing Interests Statement:

GH, DN, KN, GG, AK, and HX are employees of A2 Biotherapeutics. JY is an employee of ImmPACT-Bio. This does not alter our adherence to PLOS one policies on sharing data and materials.

Best regards,

Han Xu, Ph.D.

Sr. Director of research

A2 Biotherapeutics

**PONE-D-20-12964**
A multivariate, quantitative assay that disentangles key kinetic parameters of primary human T cell function in vitro
PLOS ONE

1. Please ensure that your manuscript meets PLOS ONE's style requirements, including those for file naming. The PLOS ONE style templates can be found at

<https://journals.plos.org/plosone/s/file?id=wjVg/PLOSOne_formatting_sample_main_body.pdf> and

<https://journals.plos.org/plosone/s/file?id=ba62/PLOSOne_formatting_sample_title_authors_affiliations.pdf>

 We have formatted the manuscript according to the guidelines.

2. Thank you for stating the following in the Competing Interests section:

"GH, DN, KN, GG, AK, and HX are employees of A2 Biotherapeutics

JY is an employee of ImmPACT-Bio."

We note that one or more of the authors are employed by a commercial company: A2 Biotherapeutics, ImmPACT-Bio.

2.1. Please provide an amended Funding Statement declaring this commercial affiliation, as well as a statement regarding the Role of Funders in your study. If the funding organization did not play a role in the study design, data collection and analysis, decision to publish, or preparation of the manuscript and only provided financial support in the form of authors' salaries and/or research materials, please review your statements relating to the author contributions, and ensure you have specifically and accurately indicated the role(s) that these authors had in your study. You can update author roles in the Author Contributions section of the online submission form.

Please also include the following statement within your amended Funding Statement.

“The funder provided support in the form of salaries for authors [insert relevant initials], but did not have any additional role in the study design, data collection and analysis, decision to publish, or preparation of the manuscript. The specific roles of these authors are articulated in the ‘author contributions’ section.”

If your commercial affiliation did play a role in your study, please state and explain this role within your updated Funding Statement.

As requested, we have added the following statement: The funder provided support in the form of salaries for authors GLH, DPN, GBG, KRN, AK, and HX, but did not have any additional role in the study design, data collection and analysis, decision to publish, or preparation of the manuscript. The specific roles of these authors are articulated in the ‘author contributions’ section.

2.2. Please also provide an updated Competing Interests Statement declaring this commercial affiliation along with any other relevant declarations relating to employment, consultancy, patents, products in development, or marketed products, etc.

Within your Competing Interests Statement, please confirm that this commercial affiliation does not alter your adherence to all PLOS ONE policies on sharing data and materials by including the following statement: "This does not alter our adherence to  PLOS ONE policies on sharing data and materials.” (as detailed online in our guide for authors http://journals.plos.org/plosone/s/competing-interests) . If this adherence statement is not accurate and there are restrictions on sharing of data and/or materials, please state these. Please note that we cannot proceed with consideration of your article until this information has been declared.

As requested, we have included the statement: "This does not alter our adherence to PLOS ONE policies on sharing data and materials.”

Please include both an updated Funding Statement and Competing Interests Statement in your cover letter. We will change the online submission form on your behalf.

Please know it is PLOS ONE policy for corresponding authors to declare, on behalf of all authors, all potential competing interests for the purposes of transparency. PLOS defines a competing interest as anything that interferes with, or could reasonably be perceived as interfering with, the full and objective presentation, peer review, editorial decision-making, or publication of research or non-research articles submitted to one of the journals. Competing interests can be financial or non-financial, professional, or personal. Competing interests can arise in relationship to an organization or another person. Please follow this link to our website for more details on competing interests: http://journals.plos.org/plosone/s/competing-interests

We have provided the amended Funding and Competing Interests statements in the cover letter per request and thank the editor in advance for altering the online submission.

Reviewers' comments:

Reviewer's Responses to Questions

**Comments to the Author**

1. Is the manuscript technically sound, and do the data support the conclusions?

The manuscript must describe a technically sound piece of scientific research with data that supports the conclusions. Experiments must have been conducted rigorously, with appropriate controls, replication, and sample sizes. The conclusions must be drawn appropriately based on the data presented.

Reviewer #1: Yes

Reviewer #2: Yes

2. Has the statistical analysis been performed appropriately and rigorously?

Reviewer #1: Yes

Reviewer #2: Yes

3. Have the authors made all data underlying the findings in their manuscript fully available?

The [PLOS Data policy](http://www.plosone.org/static/policies.action#sharing) requires authors to make all data underlying the findings described in their manuscript fully available without restriction, with rare exception (please refer to the Data Availability Statement in the manuscript PDF file). The data should be provided as part of the manuscript or its supporting information, or deposited to a public repository. For example, in addition to summary statistics, the data points behind means, medians and variance measures should be available. If there are restrictions on publicly sharing data—e.g. participant privacy or use of data from a third party—those must be specified.

Reviewer #1: Yes

Reviewer #2: Yes

4. Is the manuscript presented in an intelligible fashion and written in standard English?

PLOS ONE does not copyedit accepted manuscripts, so the language in submitted articles must be clear, correct, and unambiguous. Any typographical or grammatical errors should be corrected at revision, so please note any specific errors here.

Reviewer #1: Yes

Reviewer #2: Yes

5. Review Comments to the Author

Please use the space provided to explain your answers to the questions above. You may also include additional comments for the author, including concerns about dual publication, research ethics, or publication ethics. (Please upload your review as an attachment if it exceeds 20,000 characters)

Reviewer #1: Huang et al report a multivariate, quantitative assay to address kinetic measures of human T cells functional tests. Imagining based T cell functional test offers several advantage over classical methods. This method records time and antigen dependent T cell proliferation and cytotoxicity at several effector to target ratio. Informed consent and institutional guidelines, and HIPPA compliance were followed to conduct this study.
T2, Jurkat, and HPV-negative HLA-A2 positive target cell lines were used. Figure 1- Schema of T cell transduction defines experimental design.
Table 1: Only HLA-A2 restricted constructs are used and antigen peptides are shown. Table 2: A detail summary of measured parameters for constructs are shown.

A limitataion to Qulk assay to its inability for baseline and tonic signaling is shown. HPVE7 CAR target cell independent killing is highlighted. Supporting information is well described and included FACS data for gene expression, Confluency, T cell#, Target cell killing, and specific killing.

Overall this study is well designed and nicely written with enough details for replication purpose.

Reviewer #2: The manuscript titled “A multivariate, quantitative assay that disentangles key kinetic parameters of primary human T cell function in vitro” by Huang et al. addresses a key technical gap in the T cell field: how to separate the key functional parameters of time- and antigen- dependent T cell proliferation and cytotoxicity. This methodology will not only be useful for high content analysis of T cell function for adoptive therapies, but also for basic understanding of effector T cell biology using high-throughput assays. The authors establish a Quantitative Imaging-based Killing (QuIK) assay, where antigen-specific T cells kill rate can be distinguished from their proliferation rate. This assay revealed some important differences in cytotoxic capacity of cells bearing different antigen receptors. In my opinion, the manuscript is an important advance in the field of CAR- and TCR- T cell therapy.
**My only question is regarding one of the experiments (Figure 2B,C) where authors show that the T cell proliferation and killing are reciprocally linked, and show different values at different E:T ratios. For example, proliferation is lower at high E:T (3:1) and higher at low E:T (1:3) whereas Killing follows an opposite behavior. Are these behaviors assay dependent? Or this is also seen outside of QuIK assay. My suggestion is to measure these parameters independently to assess that, and discuss a possible explanation underlying this behavior in the Discussion section of the manuscript.**

We are very grateful to the reviewer for pointing out this inconsistency, and we have addressed it in the manuscript. We used an orthogonal method, flow cytometry, to measure proliferation (S5 Fig.). Even though it was challenging to compare among different E:T ratio groups due to T cell recovery, we demonstrated the inverse correlation between cytotoxicity and proliferation within each E:T ratio. In the meantime, we acquired a more sophisticated (and expensive) imaging instrument called the ImageXpress Micro (IXM). When we compared results between the Incucyte and IXM, we noticed that at high E:T ratios, the Incucyte systematically underestimates T cell counts, because of the tendency for T cells at high concentration to aggregate in the periphery of wells. The Incucyte can only image the well center; the IXM images the whole well. Even with this difference, we observed a similar trend to Incucyte results with slight differences: 1) the T cell proliferation was similar across different E:T ratio groups (all are very modest), with the exception that, for the 3:1 group at the highest peptide concentration, total T cell numbers decreased over time. This result does not change the fundamental conclusions. However, it does suggest that a more expensive device has advantages.

We have altered the manuscript text to reflect these conclusions; specifically, we have:

1. added a section of flow cytometry to Methods (line 111-125);
2. added a sentence in Methods (QuIK Assay section) to reflect that the experiments can be done on both the IncuCyte and IXM (line 202);
3. added the following lines in Results to include the new data:
   a. Line 296-298
   b. Line 373-383
4. changed the following in Discussion:
   a. Line 474-477
   b. Line 499-505
5. added figures S4 and S5 to show data from the IXM and flow cytometry experiments.

In addition, a minor suggestion is to change the color scheme of the traces in Figure 2B, C and Figure 3 to allow them to be more distinguishable.

We have improved the clarity by increasing the line thickness.

6. PLOS authors have the option to publish the peer review history of their article. If published, this will include your full peer review and any attached files.


If you choose “no”, your identity will remain anonymous but your review may still be made public.


**Do you want your identity to be public for this peer review?** For information about this choice, including consent withdrawal, please see our [Privacy Policy](https://www.plos.org/privacy-policy).

Reviewer #1: **Yes:** Raghvendra M. Srivastava, PhD

Reviewer #2: No
